# FEASIBILITY OF SCREENING AND MANAGING CAREGIVER BURDEN AND DEPRESSIVE SYMPTOMS DURING PATIENTS’ CLINIC VISITS

**DOI:** 10.1093/geroni/igz038.2819

**Published:** 2019-11-08

**Authors:** Ranak Trivedi, Rashmi Risbud, Manali Patel, Steven Asch, Karl Lorenz

**Affiliations:** 1 Center for Innovation to Implementation, VA Palo Alto Health Care System, Menlo Park, California, United States; 2 VA Palo Alto Health Care System, Menlo Park, California, United States; 3 Stanford University, Stanford, California, United States

## Abstract

Half of cancer caregivers experience depression, caregiver burden, or stress, yet less than a third have discussed their needs with anyone. Identifying this vulnerable population is challenging since caregivers only interact with the healthcare system in service of the patients. Our objectives were: 1) To test the feasibility of screening cancer caregivers for burden and depressive symptoms during patients’ clinic visits; and 2) To test the feasibility of a brief counseling session for caregivers who screened positive for either. Caregivers of patients with head and neck cancers were recruited from cancer clinic waiting rooms at Palo Alto VA and Stanford. Caregivers completed the PHQ-9 (depressive symptoms), and Zarit Burden Inventory-Short Form (caregiver burden). Participants screening positive for burden (>16) and/or depressive symptoms (>9) were provided psychoeducational resources and the choice to attend 1 brief counseling session with a clinical psychologist. Of the 50 participants who completed the surveys, 36 (72%) were women and 30 (60%) were significant others. Mean scores for depressive symptoms and caregiver burden were 6.29±5.01 and 11.02±8.62, respectively. Twenty participants screened positive for depressive symptoms (n=9) or caregiver burden (n=11); 3 screened positive for both. Of those who screened positive, only 4 indicated an interest in counseling. Main reason for refusal was lack of time, or that they were already receiving mental health care. Screening caregivers at patient visits is feasible and convenient. However, connecting those in need to mental health resources may be more challenging.

